# Fertility Preferences and Contraceptive Change in Low‐ and Middle‐Income Countries

**DOI:** 10.1111/sifp.12202

**Published:** 2022-06-21

**Authors:** Mobolaji Ibitoye, John B. Casterline, Chenyao Zhang

**Affiliations:** ^1^ Institute for Population Research, Ohio State University Columbus OH 43210 USA; ^2^ Department of Sociology, Ohio State University Columbus OH 43210 USA

## Abstract

The past four decades have witnessed an enormous increase in modern contraception in most low‐ and middle‐income countries. We examine the extent to which this change can be attributed to changes in fertility preferences versus fuller implementation of fertility preferences, a distinction at the heart of intense debates about the returns to investments in family planning services. We analyze national survey data from five major survey programs: World Fertility Surveys, Demographic Health Surveys, Reproductive Health Surveys, Pan‐Arab Project for Child Development or Family Health, and Multiple Indicator Cluster Surveys. We perform regression decomposition of change between successive surveys in 59 countries (330 decompositions in total). Change in preferences accounts for little of the change: less than 10 percent in a basic decomposition and about 15 percent under a more elaborate specification. This is a powerful empirical refutation of the view that contraceptive change has been driven principally by reductions in demand for children. We show that this outcome is not surprising given that the distribution of women according to fertility preferences is surprisingly stable over time.

## BACKGROUND

The increased prevalence of modern contraception in low‐ and middle‐income countries (LMIC) during the past 50 years ranks among the most important changes in the demography of these societies. The increase is relatively precisely measured because of the ubiquity of regular national demographic surveys, beginning with the World Fertility Surveys (WFS) and continuing through the Demographic Health Surveys (DHS) and Multiple Indicator Cluster Surveys (MICS), as well as other survey programs. Few demographic changes have been so well documented (United Nations Population Division n.d.).

While an increase in contraceptive prevalence per se is well documented, the source of this change is a matter of dispute. By “source,” we refer to the fundamental question of whether increased contraceptive prevalence is due to

- increased desire to avoid pregnancy (i.e., delay births or terminate childbearing) or
- increased implementation of existing desires to avoid pregnancy.

The relative weighting of these two sources of contraceptive change has been hotly debated, in both scientific and policy forums, because it speaks directly to the major policy question of the potential contribution of expanding family planning services. The debate emerged in full force in the 1960s (Davis 1967) and was the focus of the influential mid‐1990s article by Lant Pritchett (1994). Davis and Pritchett took the stance that dominates in the social science literature, namely that fertility decline is driven mainly by the decline in desired fertility, itself due to improvements in child survival and a changing balance of childbearing costs and benefits (Galor 2012). However, accumulating empirical evidence casts doubt on the sufficiency of this explanation. Most notably, there have been sharp declines in rates of unwanted fertility (Bearak et al. 2018; Casterline and El‐Zeini 2022; Sedgh, Singh, and Hussain 2014), and these have made a substantial contribution (on the order of one‐half) to contemporary fertility declines (Günther and Harttgen 2016). This empirical evidence suggests that the implementation of fertility preferences is an important driver of reproductive change.

We contribute further evidence that speaks to this key debate through the analysis of five decades of national demographic survey data (the 1970s through 2020). These surveys contain measurements of both contraceptive practice (contraception prevalence rate [CPR]) and fertility preferences (want soon, want later, want no more) at the time of the survey. Employing regression decomposition, we attribute a change in CPR between successive surveys in the same country to two components: change in fertility preferences and change in rates of contraception use within categories of fertility preferences.

A predecessor for this research is work conducted by Feyisetan and Casterline (2000). This article, published two decades ago, presented decompositions of contraceptive change in 26 countries (one decomposition per country) in the period from the mid‐1970s to the late 1990s. The decomposition results indicated that most of the increase in prevalence could be attributed to a more complete implementation of fertility preferences—at least 70 percent of the increase in 24 of the 26 countries. The present research extends the observation period by two decades—through 2020—and encompasses about twice as many countries. Also, Feyisetan and Casterline (2000) analyzed the change in CPR, whereas the present research focuses on modern methods (mCPR). (We also report results for any method.)

## METHODS

### Data

We analyze data from five major survey programs: WFS, DHS, Reproductive Health Surveys (RHS), MICS, and Pan‐Arab Project for Child Development or Family Health (PAP; a survey program conducted by the Arab League). A few surveys conducted outside these international programs are also included. All the surveys offer moderate or large nationally representative probability samples of women of reproductive age (aged 15–49).

Because this is an analysis of within‐country change, a country must offer at least two surveys. We also impose a rule that the number of years between surveys must be at least eight years and the amount of change in CPR must be at least eight percentage points. (The empirical results are not sensitive to this rule if the amount of change is at least five percentage points.) With this rule, in most countries, each decomposition is not based on successive surveys but instead skips one or more surveys. We allow for more than one decomposition per country, provided the historical difference and amount of CPR change between each pair of surveys satisfy the rule. We also exclude countries with a total population size of less than 1 million persons in the year 2000.

As explained below, fertility preference has two variants: the first is a simple dichotomy of wanting versus not wanting another child, and the second distinguishes also women who want to delay the next birth. We label the decompositions based on these two variants of the preference variable “without spacing” and “with spacing,” respectively. Some surveys only support “without spacing,” whereas others support both “without spacing” and “with spacing.”

The resulting sample of countries, surveys, and decompositions is as follows:

**Table:** 

	Without spacing	With spacing
Number of countries	59	54
Number of surveys	174	144
Number of decompositions	187	143

Regional breakdowns of the number of countries, surveys, and decompositions are shown in Table 1. A full listing of surveys, by country and survey program, is shown in the online Appendix (Table T1).

**TABLE 1:** Number of countries, surveys, and decompositions in the analyses of modern contraception, by region

Region	Number of countries	Number of surveys	Number of decompositions
**Panel a: analysis without spacing**			
East and Southern Africa	13	38	25
Middle and West Africa	14	29	15
Latin America and Caribbean	15	45	30
South and Southeast Asia	9	22	13
West Asia and North Africa	7	20	13
Total	58	154	96
**Panel b: analysis with spacing**			
East and Southern Africa	13	35	22
Middle and West Africa	14	29	15
Latin America and Caribbean	13	36	23
South and Southeast Asia	7	16	9
West Asia and North Africa	6	13	7
Total	53	129	76

The analysis depends on merely three variables: contraceptive use, fertility preferences, and parity at the time of the survey. Contraceptive use refers to the method, if any, that a woman is using at the time of the survey. We adhere to the established designation of “modern methods” (World Health Organization n.d.) except that the lactational amenorrhea method (LAM) is excluded.^1^ There is some variation across surveys in the questionnaire strategy for ascertaining current use, but there is no evidence this variation produces noncomparability in the measurement of contraceptive behavior.

The woman's fertility preference is obtained via two items. The first asks “Would you like to have (a/another) child, or would you prefer not to have any (more) children?” On this basis, women are classified as wanting or not wanting an additional child. (Women who are uncertain are grouped with the women who want another child, as uncertainty indicates an openness to having another child.) Most, but not all, surveys ask a follow‐up question to women who want another child: “How long would you like to wait from now before the birth of another child?” Women who express a wish to wait two years or longer are classified as wanting to delay the next child, yielding a three‐category preference variable: want soon, want later, do not want. Note that the well‐established global indicators “demand for family planning,” “unmet need for contraception,” and “demand satisfied” are constructed using the same fertility preference variables as used in this analysis (World Health Organization n.d.).

Pregnant women and infecund women, both small fractions of women of reproductive age, require special treatment. Pregnant women are assigned a fertility preference category based on the reported wanted status of the pregnancy (wanted then, wanted later, not wanted). In those few surveys where currently pregnant women were not asked about the wanted status of the pregnancy, we infer whether it was wanted or not by comparing the woman's number of living children with her stated ideal number of children (but we are unable to determine whether wanted pregnancies were mistimed, that is, occurred sooner than wanted). Note that pregnant women are not excluded, as is the case in some analyses of contraception. Excluding pregnant women is a form of selection on the dependent variable: currently pregnant women are selective of nonusers.

Women who self‐report as being infecund present a measurement problem. In some surveys (most notably WFS), infecund women are not asked about their fertility preferences. In many other surveys (e.g., DHS), “cannot get pregnant” is a category in the fertility preference item; it is not explicitly offered to women, but the interviewer checks this box if women volunteer that they do not believe they can conceive. For these women, it is not known whether they wanted another child or not. In short, in most surveys available for this research, fertility preferences are missing for women who self‐report as infecund. Our solution is to exclude these women entirely from the analysis; this is tantamount to an assumption that infecundity is random with respect to fertility preferences. In the surveys selected for this analysis, the fraction of women who self‐reported being infecund ranges from zero percent to nine percent, with a median of less than two percent.

In all surveys, the analysis is restricted to women currently in‐union. This restriction is applied in the interest of comparability; in a subset of surveys, contraception, and/or fertility preferences were only measured for women currently in‐union. And by usual convention, this is the subpopulation for mCPR (World Health Organization n.d.).

We also exclude women aged 45 and above. A large fraction of these women self‐report as being infecund and, in any case, the risk of conceiving is low and hence contraception is of little relevance for most of these women.

The sample for this analysis, therefore, is in‐union women aged 15–44 who do not self‐report as infecund.

### Decomposition Approach

We perform a demographic decomposition via regression decomposition, applying conventional and well‐established formulae (Fortin, Lemieux, and Firpo 2011).^2^ At the individual level, the use of contraception (dichotomy) is regressed on fertility preferences (two‐category or three‐category variable, for without spacing and with spacing variants, respectively). For each pair of surveys in the decomposition, linear regressions are estimated. On the basis of the proportions of women in each preference category and the estimated regression coefficients for each category, change in contraceptive use is attributed to (i) a change in preference composition and (ii) a change in the effects of each preference category (i.e., the regression coefficients), essentially rates of contraceptive use within fertility preference categories. Following the convention in demographic decomposition, these two components are “composition” and “rates,” respectively.

We elaborate the decomposition by including parity. Close examination of the data reveals that in many countries parity‐specific preferences shifted toward wanting to avoid pregnancy (delay next birth or have no further children), while at the same time the parity distribution shifted toward lower parities (Zhang 2019). From the standpoint of the preference composition of all in‐union reproductive‐age women, these are offsetting shifts, yielding rather minimal change in the fraction of women wanting to space or limit (as we document below—see Tables in the Results section). By incorporating parity in the decomposition (and therefore performing the decomposition with parity‐specific preferences), we allow for the stronger expression of ongoing changes in fertility preferences.^3^

**TABLE 2:** Mean percentage of currently married women aged 15–44 using modern contraception and wanting to stop childbearing by survey, percentage‐point change between surveys and per annum change, by region (without spacing)

Region	Survey 1	Survey 2	Intersurvey change	Per annum change
**Panel a: Average percentage of modern contraception**				
East and Southern Africa	21.7	41.1	19.5	1.5
Middle and West Africa	4.4	17.9	13.6	0.61
Latin America and Caribbean	41.3	56.8	15.5	1.17
South and Southeast Asia	26.8	45.5	18.7	1.21
West Asia and North Africa	25.1	40.3	15.2	1.02
Total	26.3	42.9	16.6	1.15
**Panel b: Average percentage wanting to stop childbearing**				
East and Southern Africa	29.8	37.6	7.8	0.54
Middle and West Africa	13.4	20.4	7.1	0.26
Latin America and Caribbean	51.8	55.6	3.9	0.27
South and Southeast Asia	45.2	54.1	8.9	0.57
West Asia and North Africa	40.0	47.9	7.9	0.53
Total	37.6	44.2	6.6	0.41

**TABLE 3:** Mean percentage of currently married women aged 15–44 using modern contraception and wanting to delay or stop childbearing by survey, percentage‐point change between surveys and per annum change, by region (with spacing)

Region	Survey 1	Survey 2	Intersurvey change	Per annum change
**Panel a: Average percentage of modern contraception**				
East and Southern Africa	23.3	43.2	19.8	1.64
Middle and West Africa	5.1	17.9	12.9	0.64
Latin America and Caribbean	41.8	56.6	14.8	1.19
South and Southeast Asia	29.6	44.8	15.2	0.99
West Asia and North Africa	30.3	46.6	16.3	1.04
Total	26.7	42.8	16.1	1.17
**Panel b: Average percentage wanting to delay or stop childbearing**				
East and Southern Africa	65.5	71.8	6.3	0.5
Middle and West Africa	56.2	63.1	7.0	0.29
Latin America and Caribbean	76.7	80.5	3.8	0.31
South and Southeast Asia	75.5	77.4	1.9	0.13
West Asia and North Africa	70.4	72.7	2.3	0.21
Total	68.7	73.5	4.8	0.33

A word on interpretation of the “rates” component. It would be convenient to regard this simply as the contribution to the contraceptive change of “implementation of preferences,” but this would be inaccurate. We adhere to the established convention that women who want another birth within two years—the “want soon” category—do not have a need for contraception (Bradley et al. 2012). But a small fraction of these women use contraception, and they can contribute to the increase in CPR. Unfortunately, this subset of women cannot be distinguished in the decomposition *without spacing*, and hence their portion of the rates component cannot be identified. This makes “implementation of preferences” a misleading label for the rates component in this decomposition. By contrast, this subset of women can be distinguished in the decomposition *with spacing*. In this decomposition, we reserve the label “implementation of preferences” for the portion of the rates component contributed by the two categories of women who want to delay the next birth or have no further births.

The meaningfulness of the decomposition rests on an important theoretical assumption, namely that contraception can be regarded as entirely the consequence of the preference distribution and the coefficients of each preference category. That is, there are no important confounding variables omitted from this analysis that exercise direct effects on contraceptive use (see Fortin, Lemieux, and Firpo 2011). The assumption underlying our analysis is that other variables affect contraception indirectly by affecting either preference (“composition”) or the translation of preferences into behavior (“rates”). This theoretical stance follows from the Easterlin Synthesis Framework (Easterlin 1975), in which the two direct determinants of fertility regulation behavior are “motivation” and “costs of regulation.” We regard the rates component as indicative of the costs of regulation. Hence a variable such as educational attainment—a strong correlate of contraception in almost every setting—is posited to affect contraception either by determining preference composition or by determining the rate of translation of preferences into use (or both).

The decompositions are performed using the Stata procedure “oaxaca” (Jann 2008). The decomposition outcome is expressed as three percentage contributions to change in contraception, summing to 100 percent: a “composition” component (distribution according to fertility preferences); a “rates” component (rates of use within preference categories, as represented by the regression coefficients for each category); and an “interaction” component, which is a residual. As shown in Results, the interaction component is very small on average (less than three percent) and is larger than 10 percent in absolute value (i.e., negative or positive) in 17 percent of the decompositions (under our most stringent specifications).

When regression decomposition employs categorical predictor variables, such as fertility preferences, an “identification problem” presents itself. To resolve this, “oaxaca” adopts the “normalization” approach proposed by Yun (2005, 2008).

Our analytic approach yields four sets of results: (i) without spacing and not controlling for parity, (ii) without spacing and controlling for parity, (iii) with spacing and not controlling for parity, and (iv) with spacing and controlling for parity. We present the findings of the analysis of change in modern methods below. The analysis of all methods yielded similar results; these are shown in the online Appendix.

## RESULTS

### Change in Modern Contraception

The average mCPR at each survey is presented by region in Tables 2 and 3. Overall, modern contraception increased by an average of about 16 percentage points between surveys (in both analyses, without (Table 2, panel a) and with spacing (Table 3, panel a). Middle and West Africa had the lowest mCPR at each survey and experienced the least amount of change between surveys.

### Change in Fertility Preferences

In the analysis without spacing, the percentage of women wanting to stop childbearing increased by almost seven percentage points on average between surveys (Table 2, panel b). Latin America and the Caribbean experienced the least amount of change. The rate of change is 0.41 percentage points per annum. This contrasts with the far more rapid change in mCPR of 1.15 percentage points per annum (Table 2, panel a). In the analysis with spacing, the percentage of women wanting to delay or stop childbearing increased by an average of five percentage points between surveys (Table 3, panel b). South and Southeast Asia experienced the least amount of change on average primarily due to a decline in the percentage of women wanting to delay childbearing between surveys. The per annum rate of change is 0.33 percentage points, in contrast to the 1.17 percentage point rate of change in mCPR.

**FIGURE 1:**
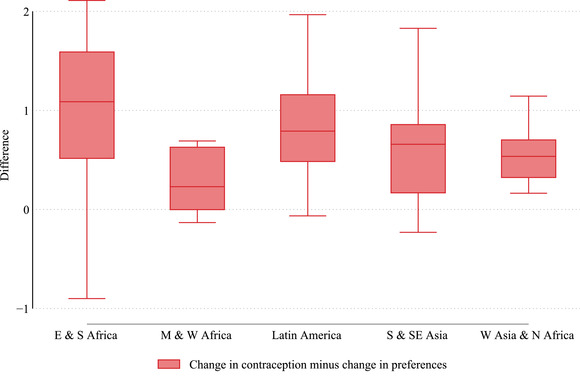
Difference in per annum change in modern contraception versus preferences (without spacing)

**FIGURE 2:**
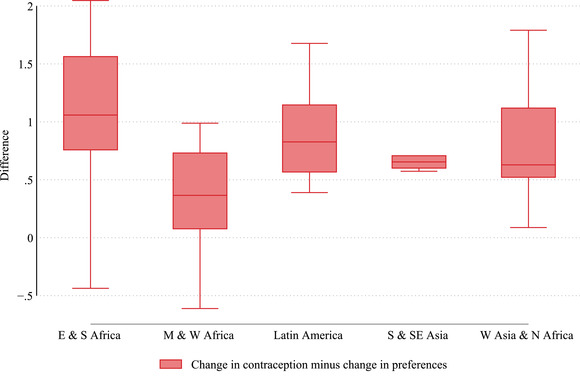
Difference in per annum change in modern contraception versus preferences (with spacing)

The fact that the per annum rate of change in mCPR generally outpaces that of fertility preferences is plainly evident in the decomposition‐by‐decomposition displays in Figures 1 and 2. The per annum rate of change in mCPR was greater than that of fertility preferences in over 86 percent of the decompositions in the analysis without spacing. Notable exceptions to this are Kenya (1978–1989) and Namibia (1992–2000), both of which experienced about a three percent per annum rate of change in the desire to stop childbearing in the noted time periods. Fertility preferences changed at a slower rate in both countries in subsequent intersurvey periods.^4^ In the analysis with spacing, the per annum rate of change in mCPR outpaced that of fertility preferences in over 96 percent of the decompositions.

### Decomposition of Change in Modern Contraception

We begin by considering how much of the mCPR change can be attributed to changes in fertility preferences (“composition”). The striking result is the small size of this component: less than 10 percent of the change in mCPR on average not accounting for parity‐specific preferences (Tables 4 and 5, panel a), and about 15 percent when parity‐specific preferences are accounted for (Tables 4 and 5, panel b). This outcome hardly differs whether spacing preferences are or are not considered (Table 4 vs. Table 5).

**TABLE 4:** Decomposition of change in mCPR: mean percentage distribution of change in mCPR, by region (without spacing)

Region	Composition	Interaction	Rates
**Panel a: Not controlling for parity**			
East and Southern Africa	5.8	1.8	92.4
Middle and West Africa	4.0	2.3	93.7
Latin America and the Caribbean	4.9	1.8	93.3
South and Southeast Asia	13.6	3.6	82.8
West Asia and North Africa	15.1	2.2	82.7
Total	7.5	2.2	90.3
**Panel b: Controlling for parity**			
East and Southern Africa	9.3	3.3	87.5
Middle and West Africa	5.0	2.3	92.6
Latin America and Caribbean	15.7	3.0	81.3
South and Southeast Asia	21.8	4.6	73.6
West Asia and North Africa	23.3	0.4	76.3
Total	14.2	2.8	83.0

**TABLE 5:** Decomposition of change in mCPR: mean percentage distribution of change in mCPR, by region (with spacing)

Region	Composition	Interaction	Rates			
			Overall	Want soon	Want later	Do not want
**Panel a: Not controlling for parity**						
East and Southern Africa	8.2	3.0	88.8	13.8	42.0	33.0
Middle and West Africa	5.3	5.6	89.1	8.0	47.0	34.1
Latin America and Caribbean	9.9	2.7	87.5	20.7	36.8	30.0
South and Southeast Asia	12.9	‐1.4	88.6	15.5	44.8	28.3
West Asia and North Africa	9.0	‐0.6	91.6	14.2	41.6	35.8
Total	8.8	2.6	88.7	15.0	41.7	32.0
**Panel b: Controlling for parity**						
East and Southern Africa	12.3	4.3	83.4	18.6	35.8	29.0
Middle and West Africa	6.3	6.2	87.6	15.7	40.6	31.3
Latin America and Caribbean	19.5	2.5	78.0	20.9	30.4	26.7
South and Southeast Asia	24.5	‐1.7	77.2	17.3	35.5	24.5
West Asia and North Africa	20.7	‐0.7	80.0	18.3	31.7	30.0
Total	15.5	2.9	81.5	18.5	34.7	28.3

In contrast, change in the rates of contraceptive use within fertility preference categories (“rates”) contributes about 90 percent of the change in mCPR on average (Tables 4 and 5) in the basic decomposition that does not account for parity. Accounting for parity‐specific preferences, the “rates” component drops to a bit more than 80 percent on average. In the analysis with spacing, we can confine the contribution of increased implementation of fertility preferences to those women who want to delay or stop childbearing, setting aside increased use among women without a need for contraception according to the standard convention (those who want a child soon, i.e. within two years); this reduces this contribution to 74 percent of change in mCPR on average not accounting for parity and 63 percent when parity‐specific preferences are accounted for (Table 5). Interestingly, increased rates of contraceptive use among women who want a child soon also account for a nontrivial proportion of the change in mCPR (15–18.5 percent on average). This subset of women is commonly neglected.

**FIGURE 3:**
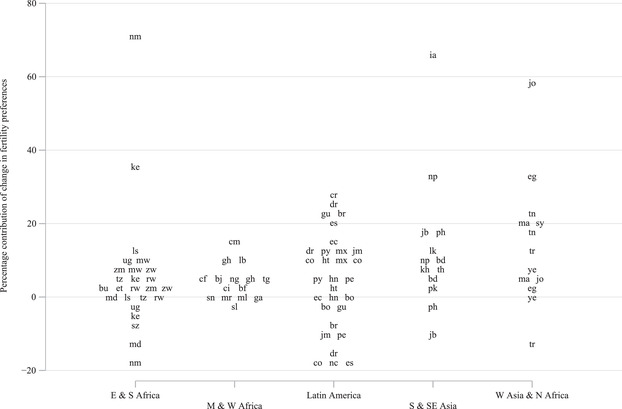
Contribution of preferences (without spacing) to change in modern contraception

**FIGURE 4:**
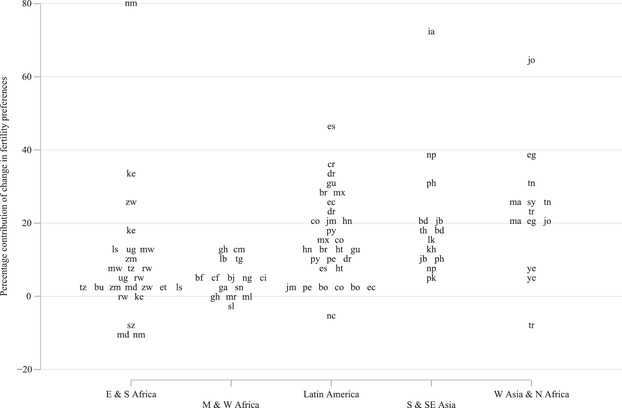
Contribution of parity‐specific preferences (without spacing) to change in modern contraception

**FIGURE 5:**
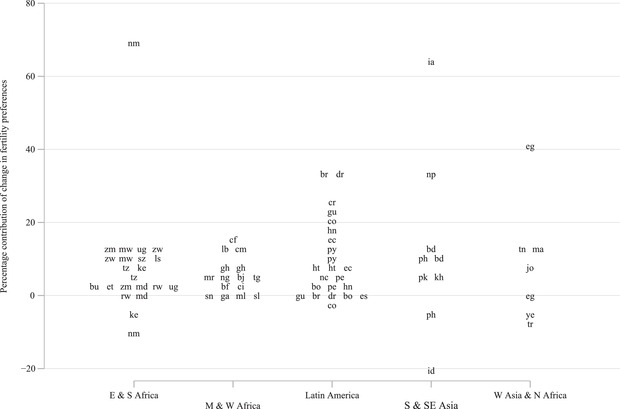
Contribution of preferences (with spacing) to change in modern contraception

**FIGURE 6:**
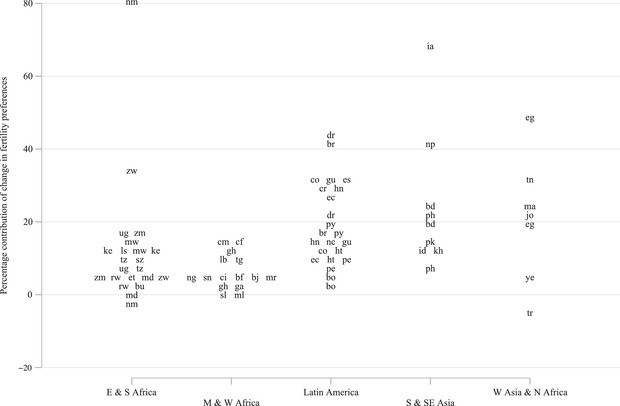
Contribution of parity‐specific preferences (with spacing) to change in modern contraception

The examination of the decomposition‐by‐decomposition results underscores the limited contribution of preference change. The values of this component for each decomposition are displayed in Figures 3, 4, 5, 6. Note that negative values of the preference composition component occur; these are instances in which fertility preferences changed in a direction that in itself would have produced a decline in mCPR. Such shifts in preference composition are moderately common, as evident in the analysis in the previous section. Without spacing (Figures 3 and 4), in more than four‐fifths of the decompositions, the composition component accounts for less than 20 percent of change in mCPR (less than 30 percent controlling for parity). A notable exception to this overall pattern is India, where the change in preferences accounts for more than 60 percent of mCPR change. From a comparative perspective, the reproductive regime that emerged in India in the 1990s and 2000s is unusual: relatively early marriage, little contraceptive practice for birth‐spacing purposes (and hence closely spaced births), and widespread adoption of sterilization after two or three births (Padmadas, Hutter, and Willekens 2004; Singh, Singh, and Singh 2021). One consequence of the shift toward this regime was a rapid increase in the proportion of in‐union women who did not want another child (sterilization locks women into the status permanently). This in turn accounts for the unusually large composition component in the decomposition for India (without spacing, and with spacing as well). Two other countries that show a large composition component are Kenya and Namibia; we have already provided some discussion of the rapid change in fertility preferences during the 1980s and 1990s, respectively, in these two countries.

The analysis with spacing yields similar patterns (Figures 5 and 6). In more than four‐fifths of the decompositions, change in preferences accounts for less than 20 percent of the change in mCPR not accounting for parity and less than 30 percent controlling for parity.

## DISCUSSION

This research addresses a large and fundamental question: To what extent can the substantial increases in contraception in recent decades in LMICs be attributed to more demand versus increased satisfaction of demand? Our results answer this question decisively and overwhelmingly against more demand: less than 10 percent of the mCPR change can be attributed to change in fertility preferences (and about 15 percent in a more elaborate decomposition that examines preferences on a parity‐specific basis). This conclusion applies to all regions—we observe no “African exceptionalism.” And only a few countries depart meaningfully from this conclusion, with India the notable and, because of its demographic weight, important exception.

We have also demonstrated that this result should occasion no surprise once one recognizes that demand, operationalized as the distribution of reproductive‐age women according to fertility preferences, has increased slowly in recent decades. The rate of increase in the percentage of women who want to avoid pregnancy (delay or stop) is about one‐quarter to one‐third of the rate of increase in the percentage of women using modern contraception. It follows that an increase in demand, as conventionally operationalized, cannot account for most of the contraceptive increase. In view of the documented declines in the desired number of children, the relative stability of fertility preference composition is a puzzle that merits its own demographic analysis. Elsewhere, we have examined this phenomenon in more depth (Zhang 2019). Declines in the desired number of children mean that women reach their desired number at a lower parity and hence, ceteris paribus, should spend a larger fraction of their reproductive years wanting to avoid pregnancy. But as it happens, several factors have acted to delay women reaching their desired number of children, specifically later age at first birth and longer interbirth spacing. From the standpoint of the overall prevalence of the preference to have no further children, these trends offset declines in desired fertility. A further factor in some instances has been shifts in the age structure toward younger women; this too acts against an increase in the fraction of women who have reached their desired number of children. Consistent with this expectation, Zhang (2019) shows that parity‐specific percentages wanting to stop or space childbearing have increased rather rapidly, but the overall prevalence of the desire to stop has changed more slowly (or not at all) due to the offsetting changes just discussed.

In asserting that change in fertility preferences has contributed minimally to contraceptive change, we must acknowledge that this is based on a simplistic measurement of preferences. Women are categorized as wanting a child soon (within two years), later, or not at all. There is no allowance for variation in motivation within these categories. But surely within these categories, women vary in the intensity of their conviction about the stated reproductive goal, for example, their conviction that they should have no further births. It is plausible that over time the composition shifts within categories toward a larger fraction of women with a strong attachment to delaying or avoiding another birth. This would constitute a genuine change in fertility preferences that is missed in our analysis, yielding an underestimate of the contribution of preference change to contraceptive change. This said, we note again that the preference indicator employed in this analysis serves as the basis for the major family planning global indicators (including demand, unmet need, and demand satisfied) (World Health Organization n.d.).

It is also important to recognize that our analysis does not speak directly to the question of to what extent increased availability of contraception, due to family planning programs and other developments, has contributed to the increased implementation of fertility preferences. Incomplete implementation can be the consequence of a host of “costs of fertility regulation” (Campbell, Sahin‐Hodoglugil, and Potts 2006; Casterline and Sinding 2000; Easterlin 1975). The costs go well beyond access to affordable family planning to include factors such as fear of health side effects, opposition from the husband and other influential persons, and concerns about the moral acceptability of contraception. Such non‐access costs can be reduced through information and services provided by family planning programs, but no doubt many non‐program factors also contribute to the decline in non‐access costs.

## CONCLUSION

The main conclusion from this research—that implementation of preferences, not change in preferences, has been the main source of contraceptive change—was obtained by means of a relatively straightforward decomposition exercise. As such, the conclusion stands as an elementary description of the nature of contraceptive change in 59 Asian, African, and Latin American countries in the decades since the 1970s. By demonstrating that considerable gains in contraceptive prevalence can be achieved simply by enabling couples to carry through on their desires to avoid pregnancy, these results clearly are more compatible with supply‐side theory than with demand‐side theory. We have provided further empirical verification of the premise that has justified the investment in family planning services in many countries during the past five decades, namely that unsatisfied demand for fertility regulation is widespread (Alkema et al. 2013; Cahill et al. 2018; Kantorová et al. 2020) and that satisfying this demand is largely responsible for driving the increases in contraceptive prevalence.

## Supporting information

TABLE T1 National demographic surveys, by region, country, year, and survey program
TABLE T2 Number of countries, surveys, and decompositions in the analyses of all contraception methods, by region
TABLE T3 Mean percentage of currently married women aged 15‐44 using any contraception and wanting to stop childbearing by survey, percentage‐point change between surveys, and per annum change, by region (without spacing)
TABLE T4 Mean percentage of currently married women aged 15‐44 using any contraception and wanting to stop childbearing by survey, percentage‐point change between surveys and per annum change, by region (with spacing)
TABLE T5 Decomposition of change in CPR: mean percentage distribution of change in CPR, by region (without spacing)
TABLE T6 Decomposition of change in CPR: mean percentage distribution of change in CPR, by region (with spacing)
FIGURE A1 Difference in per annum change in any contraception versus preferences (without spacing)
FIGURE A2 Difference in per annum change in any contraception versus preferences (with spacing)
FIGURE A3 Contribution of preferences (without spacing) to change in any contraception
FIGURE A4 Contribution of parity‐specific preferences (without spacing) to change in any contraception
FIGURE A5 Contribution of preferences (with spacing) to change in any contraception
FIGURE A6 Contribution of parity‐specific preferences (with spacing) to change in any contraception

## Data Availability

Most of the data that support the findings of this study were derived from the following resources available in the public domain: DHS & WFS ‐ https://dhsprogram.com/data/; MICS ‐ https://mics.unicef.org/surveys; RHS ‐ http://ghdx.healthdata.org/series/reproductive‐health‐survey‐rhs. The Stata programs and analytic dataset will be available from the corresponding author on reasonable request.

## Footnotes

1 Examination of within‐country trends in LAM revealed marked volatility from survey‐to‐survey in a few countries that almost certainly reflects inconsistent measurement. This in turn led to some eccentric decomposition results. Note that LAM is a tiny fraction of mCPR in the vast majority of surveys.

2 A conventional demographic decomposition yields identical results.

3 We control for parity when carrying out the decomposition by fixing the parity distribution at the first survey values. We thank John Cleland for suggesting this elaboration of the decomposition analysis.

4 The sharp shift in fertility desires in Kenya during the 1980s was noted by many analysts at the time, for example, Robinson (1992) and Sinding (1991). In the case of Namibia, we are not aware of any critical analysis of the substantial increase in stated desire to have no further children that occurred between the 1992 DHS and 2000 DHS (almost 20 percentage points in merely eight years). We have looked closely at the pertinent variables in the two files and see no obvious problems or non‐comparabilities.
