# Supplementary material for: Fertility Preferences and Contraceptive Change in Low‐ and Middle‐Income Countries
Source: Stud Fam Plann. 2022 Jun 21;53(2):361–76. doi: 10.1111/sifp.12202 (PMC9219575; doi:10.1111/sifp.12202)
Supplement: Supplementary file 1 — TABLE T1 National demographic surveys, by region, country, year, and survey program TABLE T2 Number of countries, surveys, and decompositions in the analyses of all contraception methods, by region TABLE T3 Mean percentage of currently married women aged 15‐44 using any contraception and wanting to stop childbearing by survey, percentage‐point change between surveys, and per annum change, by region (without spacing) TABLE T4 Mean percentage of currently married women aged 15‐44 using any contraception and wanting to stop childbearing by survey, percentage‐point change between surveys and per annum change, by region (with spacing) TABLE T5 Decomposition of change in CPR: mean percentage distribution of change in CPR, by region (without spacing) TABLE T6 Decomposition of change in CPR: mean percentage distribution of change in CPR, by region (with spacing) FIGURE A1 Difference in per annum change in any contraception versus preferences (without spacing) FIGURE A2 Difference in per annum change in any contraception versus preferences (with spacing) FIGURE A3 Contribution of preferences (without spacing) to change in any contraception FIGURE A4 Contribution of parity‐specific preferences (without spacing) to change in any contraception FIGURE A5 Contribution of preferences (with spacing) to change in any contraception FIGURE A6 Contribution of parity‐specific preferences (with spacing) to change in any contraception [file SIFP-53-361-s001.docx]

**APPENDIX**

**Table T1 National demographic surveys, by region, country, year, and survey program**

| **Region** | **Country** | **Country code** | **Year of Survey** |
| --- | --- | --- | --- |
| East &  South Africa | Burundi | bu | 1987, 2016 |
|  | Ethiopia | et | 2000, 2016 |
|  | Kenya | ke | 1978^W^, 1989, 1998, 2003, 2014 |
|  | Lesotho | ls | 1977^W^, 2004, 2009, 2018^M^ |
|  | Madagascar | md | 1992, 2003, 2008, 2018^M^ |
|  | Malawi | mw | 1992, 2004, 2015 |
|  | Namibia | nm | 1992, 2000, 2013 |
|  | Rwanda | rw | 1983^W^, 1992, 2000, 2010, 2020 |
|  | Eswatini | sz | 2006, 2014^M^ |
|  | Tanzania | tz | 1991, 2004, 2015 |
|  | Uganda | ug | 1988, 2006, 2016 |
|  | Zambia | zm | 1992, 2007, 2018 |
|  | Zimbabwe | zw | 1988, 1999, 2015 |
| Middle &  West Africa | Benin | bj | 1981^W^, 2018 |
|  | Burkina Faso | bf | 1998, 2010 |
|  | Cameroon | cm | 1991, 2004, 2014^M^, 2018 |
|  | Central African Republic | cf | 1994, 2019^M^ |
|  | Cote d’Ivoire | ci | 1980^W^, 1994, 2012, 2016^M^ |
|  | Gabon | ga | 2000, 2012 |
|  | Ghana | gh | 1979^W^, 1988, 1998, 2017^M^ |
|  | Liberia | lb | 1986, 2019 |
|  | Mali | ml | 1987, 2018 |
|  | Mauritania | mr | 1981^W^, 2000, 2015^M^ |
|  | Niger | ni | 1992, 2012 |
|  | Nigeria | ng | 1982^W^, 2018 |
|  | Senegal | sn | 1986, 1992, 2019 |
|  | Sierra Leone | sl | 2008, 2019 |
|  | Togo | tg | 1988, 2017^M^ |
| Latin America | Bolivia | bo | 1989, 1998, 2008 |
|  | Brazil | br | 1986, 1996, 2006^O^ |
|  | Colombia | co | 1976^W^, 1986, , 2005, 2015 |
|  | Costa Rica | cr | 1976^W^, 1986, 1999^O^ |
|  | Dominican Republic | dr | 1975^W^, 1980, 1986, 1991,1996, 2013 |
|  | Ecuador | ec | 1979^W^, 1994^R^, 2004^R^ |
|  | El Salvador | es | 1985, 1998^R^, 2008^R^, 2014^M^ |
|  | Guatemala | gu | 1987, 1995, 2015 |
|  | Haiti | ht | 1977^W^, 1994, 2005, 2017 |
|  | Honduras | hn | 1991^R^, 1996^R^, 2001^R^, 2005^R^, 2012, 2019^M^ |
|  | Jamaica | jm | 1975^W^, 1989^R^, 2008^R^ |
|  | Mexico | mx | 1976^W^, 1987, 2009^O^, 2014^O^ |
|  | Nicaragua | nc | 1998, 2006^R^ |
|  | Paraguay | py | 1979^W^, 1995^R^, 2008^R^ |
|  | Peru | pe | 1977^W^, 1986, 1996, 2012 |
| South &  Southeast Asia | Bangladesh | bd | 1989^O^, 1997, 2019^M^ |
|  | Cambodia | kh | 2000, 2014 |
|  | India | ia | 1993, 2006 |
|  | Indonesia | jb, id | 1976^W^, 1987, 1991, 2012, 2017 |
|  | Nepal | np | 1976^W^, 1996, 2001, 2016, 2019^M^ |
|  | Philippines | ph | 1978^W^, 1986^O^, 1993, 1998, 2003, 2013, 2017 |
|  | Pakistan | pk | 1975^W^, 1991, 2012, 2018 |
|  | Sri Lanka | lk | 1975^W^, 1987 |
|  | Thailand | th | 1975^W^, 1987 |
| West Asia &  North Africa | Egypt | eg | 1980^W^, 1988, 2008 |
|  | Jordan | jo | 1975^W^, 1990, 1997, 2012 |
|  | Morocco | ma | 1980^W^, 1987, 1992, 2003 |
|  | Syria | sy | 1978^W^, 2001^P^ |
|  | Tunisia | tn | 1978^W^, 1988, 2011^M^ |
|  | Turkey | tr | 1978^W^, 1993, 1998, 2013, 2018^O^ |
|  | Yemen | ye | 1979^W^, 1991, 2003^P^, 2013 |

All surveys are DHS unless otherwise indicated.

^W^ World Fertility Surveys (WFS)

^P^ Pan Arab Project for child development or family health (PAP)

^M^ Multiple Indicator Cluster Surveys (MICS)

^R^ Reproductive Health Surveys (RHS)

^O^ Other national demographic surveys

**TABLE T2 Number of countries, surveys and decompositions in the analyses of all contraception methods, by region**

| **Panel A: Analysis without spacing** | | | |
| --- | --- | --- | --- |
| **Region** | **Number of countries** | **Number of surveys** | **Number of decompositions** |
| East & Southern Africa | 13 | 38 | 25 |
| Middle & West Africa | 10 | 21 | 11 |
| Latin America & Caribbean | 15 | 43 | 28 |
| South & Southeast Asia | 9 | 22 | 13 |
| West Asia & North Africa | 7 | 21 | 14 |
| Total | 54 | 145 | 91 |
| **Panel B: Analysis with spacing** | | | |
| **Region** | **Number of countries** | **Number of surveys** | **Number of decompositions** |
| East & Southern Africa | 13 | 36 | 22 |
| Middle & West Africa | 9 | 20 | 10 |
| Latin America & Caribbean | 13 | 33 | 20 |
| South & Southeast Asia | 7 | 15 | 8 |
| West Asia & North Africa | 6 | 13 | 7 |
| Total | 48 | 117 | 67 |

**TABLE T3 Mean percentage of currently married women aged 15-44 using any contraception and wanting to stop childbearing by survey, percentage-point change between surveys and per annum change, by region (without spacing)**

| **Panel a: average % any contraception** | | | | |
| --- | --- | --- | --- | --- |
| **Region** | **Survey 1** | **Survey 2** | **Inter-survey change** | **Per annum change** |
| East & Southern Africa | 27.1 | 45.5 | 18.4 | 1.41 |
| Middle & West Africa | 8.6 | 23.2 | 14.6 | 0.64 |
| Latin America & Caribbean | 50.4 | 67.1 | 16.6 | 1.30 |
| South & Southeast Asia | 34.7 | 55.6 | 20.9 | 1.38 |
| West Asia & North Africa | 33.3 | 50.8 | 17.5 | 1.21 |
| Total | 34.1 | 51.7 | 17.6 | 1.25 |
| **Panel b: average % wanting to stop childbearing** | | | | |
| **Region** | **Survey 1** | **Survey 2** | **Inter-survey change** | **Per annum change** |
| East & Southern Africa | 29.8 | 37.6 | 7.8 | 0.55 |
| Middle & West Africa | 11.1 | 18.5 | 7.4 | 0.23 |
| Latin America & Caribbean | 52.1 | 55.9 | 3.8 | 0.29 |
| South & Southeast Asia | 45.8 | 55.2 | 9.5 | 0.61 |
| West Asia & North Africa | 39.1 | 46.1 | 7.0 | 0.51 |
| Total | 38.1 | 44.8 | 6.7 | 0.43 |

**TABLE T4 Mean percentage of currently married women aged 15-44 using any contraception and wanting to stop childbearing by survey, percentage-point change between surveys and per annum change, by region (with spacing)**

| **Panel a: average % any contraception** | | | | |
| --- | --- | --- | --- | --- |
| **Region** | **Survey 1** | **Survey 2** | **Inter-survey change** | **Per annum change** |
| East & Southern Africa | 28.9 | 47.2 | 18.3 | 1.50 |
| Middle & West Africa | 10.0 | 23.7 | 13.7 | 0.67 |
| Latin America & Caribbean | 51.1 | 68.2 | 17.1 | 1.27 |
| South & Southeast Asia | 35.7 | 55.9 | 20.2 | 1.29 |
| West Asia & North Africa | 40.8 | 58.8 | 18.0 | 1.14 |
| Total | 34.8 | 52.2 | 17.4 | 1.24 |
| **Panel b: average % wanting to delay or stop childbearing** | | | | |
| **Region** | **Survey 1** | **Survey 2** | **Inter-survey change** | **Per annum change** |
| East & Southern Africa | 65.3 | 71.5 | 6.3 | 0.50 |
| Middle & West Africa | 52.3 | 59.8 | 7.6 | 0.36 |
| Latin America & Caribbean | 76.4 | 80.5 | 4.1 | 0.31 |
| South & Southeast Asia | 75.6 | 77.2 | 1.6 | 0.13 |
| West Asia & North Africa | 70.4 | 72.4 | 2.0 | 0.19 |
| Total | 68.4 | 73.2 | 4.8 | 0.35 |

**FIGURE A1 Difference in per annum change in any contraception versus preferences (without spacing)**

**FIGURE A2 Difference in per annum change in any contraception versus preferences (with spacing)**

**TABLE T5 Decomposition of Change in CPR: Mean Percentage Distribution of Change in CPR, by Region (Without Spacing)**

| **Panel a: Not controlling for parity** | | | |
| --- | --- | --- | --- |
| **Region** | **Composition** | **Interaction** | **Rates** |
| East & Southern Africa | 7.2 | 1.2 | 91.6 |
| Middle & West Africa | 6.6 | 0.1 | 93.4 |
| Latin America & Caribbean | 6.5 | 0.8 | 92.7 |
| South & Southeast Asia | 15.1 | 2.7 | 82.2 |
| West Asia & North Africa | 11.1 | 2.1 | 86.9 |
| Total | 8.6 | 1.3 | 90.1 |
|  |  |  |  |
| **Panel b: Controlling for parity** | | | |
| **Region** | **Composition** | **Interaction** | **Rates** |
| East & Southern Africa | 11.2 | 2.6 | 86.2 |
| Middle & West Africa | 7.9 | -0.2 | 92.3 |
| Latin America & Caribbean | 17.9 | 1.7 | 80.4 |
| South & Southeast Asia | 25.2 | 2.9 | 71.9 |
| West Asia & North Africa | 20.4 | -0.3 | 79.8 |
| Total | 16.3 | 1.6 | 82.1 |

**TABLE T6 Decomposition of Change in CPR: Mean Percentage Distribution of Change in CPR, by Region (With Spacing)**

| **Panel A: Not controlling for parity** | | | | | | |
| --- | --- | --- | --- | --- | --- | --- |
| **Region** | **Composition** | **Interaction** | **Rates** | | | |
|  |  |  | **Overall** | **Want more soon** | **Want later** | **Do not want** |
| East & Southern Africa | 11.1 | 2.3 | 86.6 | 13.8 | 41.7 | 31.1 |
| Middle & West Africa | 9.8 | 5.3 | 84.9 | 2.4 | 49.9 | 32.6 |
| Latin America & Caribbean | 12.9 | 2.2 | 85.0 | 22.5 | 35.1 | 27.4 |
| South & Southeast Asia | 12.0 | -1.8 | 89.8 | 15.8 | 45.2 | 28.9 |
| West Asia & North Africa | 4.9 | -0.4 | 95.4 | 17.6 | 47.3 | 30.6 |
| Total | 10.9 | 1.9 | 87.2 | 15.3 | 41.9 | 29.9 |
| **Panel B: Controlling for parity** | | | | | | |
| **Region** | **Composition** | **Interaction** | **Rates** | | | |
|  |  |  | **Overall** | **Want soon** | **Want later** | **Do not want** |
| East & Southern Africa | 16.1 | 3.4 | 80.5 | 18.3 | 35.0 | 27.3 |
| Middle & West Africa | 10.6 | 5.8 | 83.6 | 11.7 | 42.1 | 29.9 |
| Latin America & Caribbean | 23.0 | 2.8 | 74.2 | 22.8 | 26.9 | 24.5 |
| South & Southeast Asia | 23.6 | -2.3 | 78.6 | 17.9 | 35.3 | 25.5 |
| West Asia & North Africa | 19.4 | -2.1 | 82.8 | 20.7 | 36.5 | 25.6 |
| Total | 18.6 | 2.3 | 79.1 | 18.8 | 33.8 | 26.4 |

**FIGURE A3 Contribution of preferences (without spacing) to change in any contraception**

**FIGURE A4 Contribution of parity-specific preferences (without spacing) to change in any contraception**

**FIGURE A5 Contribution of preferences (with spacing) to change in any contraception**

**FIGURE A6 Contribution of parity-specific preferences (with spacing) to change in any contraception**
